# Valganciclovir Underdosing Is Associated With Cytomegalovirus DNAemia During Universal Prophylaxis: A Real‐Life Case Control Retrospective Study

**DOI:** 10.1002/jmv.70666

**Published:** 2025-10-24

**Authors:** Pierre‐Antonin Rigon, Justine Solignac, Christine Zandotti, Marion Gully, Philippe Brunet, Noémie Resseguier, Tristan Legris, Valérie Moal

**Affiliations:** ^1^ Assistance Publique‐Hôpitaux de Marseille, Hôpital Conception, Centre de Néphrologie et Transplantation Rénale Marseille France; ^2^ Aix‐Marseille Université Marseille France; ^3^ Assistance Publique‐Hôpitaux de Marseille, Service de Virologie Aiguë et Tropicale Marseille France; ^4^ CEReSS/UR 3279—Health Services and Quality of Life Research Aix Marseille University Marseille France; ^5^ Methodological Support Unit for Clinical and Epidemiological Research University Hospital of Marseille Marseille France

**Keywords:** breakthrough cytomegalovirus infection, CMV, cytomegalovirus, kidney transplantation, prophylaxis, valganciclovir

## Abstract

Universal prophylaxis or a preemptive strategy with valganciclovir (VGCV) is recommended for prevention of cytomegalovirus (CMV) infections after kidney transplantation. We combined both strategies during the first 3 months post‐transplantation. The objectives were to define the incidence of CMV DNAemia and identify the associated factors during universal prophylaxis in a retrospective monocentric case‐control study. Cases presenting CMV DNAemia during the first 3 months post‐transplantation were matched to four controls each regarding sex, age, donor and recipient CMV serostatus, and induction treatment. We collected the rates of visits with VGCV underdosing, comparing the adjusted daily dosage to the kidney function and the received daily dosage. Despite prophylaxis, the incidence of CMV DNAemia was 5.1% between 2017 and 2020 in 300 patients. Rates of visits with VGCV underdosing during follow‐up were significantly higher in the cases group than in the controls group. In the cases group, the rate was higher when using estimated creatinine clearance rather than estimated glomerular filtration rate. VGCV underdosing was the only factor associated with CMV DNAemia in the multivariate analysis (OR: 1.04 95% CI: 1.011–1.061, *p* = 0.003), regardless of the formula used to estimate kidney function. Resistance to GCV was observed in 4 out of 15 cases, two of which had VGCV underdosing. In our study, CMV DNAemia was associated with VGCV underdosing that could be related to the absence of systematic adjustment during post‐transplantation follow‐up or to the use of inadequate kidney function assessment formulas to adjust VGCV dosages.

## 1 Introduction

There have been major advances in the prevention and treatment of cytomegalovirus (CMV) infections in the past decades but these remain among the commonest opportunistic infections after kidney transplantation (KT), creating additional challenges because of the rise of refractory and resistant infections, and the toxicities associated with current anti‐CMV drugs [1, 2].

The risk of CMV infection or disease after KT is determined primarily by the donor and recipient CMV IgG serostatus and can be stratified from lowest to highest: seronegative donor (D−)/recipient (R−), seropositive donor (D+)/recipient (R+), D−/R+, and D+/R− [3]. With the current preventive strategies, the incidence of CMV infections is about 17%–37%, with the highest risk in the first 100 days [3, 4].

Currently, two key strategies are used to prevent CMV infections in KT recipients (KTR)—universal prophylaxis and preemptive therapy. Universal prophylaxis involves the administration of an antiviral drug, typically valganciclovir (VGCV), to KTR with high and intermediate CMV infection risk. The preemptive strategy involves regular monitoring of blood CMV PCR and initiation of preemptive treatment when CMV DNA is detected in the blood (DNAemia). In the Fourth International Consensus Guidelines on the Management of Cytomegalovirus in Solid‐organ Transplantation, both strategies are suggested but universal prophylaxis is preferred for patients receiving induction with lymphocyte‐depleting agents [4]. However, under universal prophylaxis that does not monitor blood CMV PCR, there have been reports of cases of early CMV infections [5, 6, 7].

The choice of preventive strategy differs in each transplant center. A European survey performed in 2016 in solid organ transplant centers showed significant differences in CMV prophylaxis management [8]. Most transplant programs reported performing CMV DNA load surveillance while patients were on antiviral prophylaxis [8, 9], as was the case in the kidney transplant unit of Marseille University Hospital, from which these study patients were drawn.

In this study, we aimed to define the incidence of CMV DNAemia after KT with VGCV universal prophylaxis, to identify the associated factors and their consequences.

## 2 Patients and Methods

### 2.1 Institutional Protocols

Immunosuppressive and prophylactic treatments, as well as clinical, biological, and virological follow‐ups, were standardized throughout the study, in accordance with the usual practices of the Centre de Néphrologie et Transplantation Rénale in Marseille University Hospital. Immunosuppression comprised both induction and maintenance treatment consisting of triple therapy with a calcineurin inhibitor (CNI), antimetabolite, and prednisone. All KTR received anti‐pneumocystosis prophylaxis at least 6 months after KT. Except for D−/R− patients, every KTR received VGCV prophylaxis, starting within the first week after KT. Prophylaxis duration was 3 months in R+ (intermediate risk) and 6 months in D+/R− (high risk) KTR. In accordance with the manufacturer's protocol and international guidelines, the initial dosage of prophylactic treatment with VGCV and at each follow‐up visit was to be adjusted to the estimated creatinine clearance calculated using the Cockcroft–Gault (CG) formula, while the hospital laboratory provided the estimated glomerular filtration rate (eGFR) using the 2009 Chronic Kidney Disease Epidemiology (CKD EPI) equation [4]. The maximum prophylactic dose for patients with an estimated creatinine clearance of 60 mL/min or greater was 900 mg daily [4].

After discharge from hospital, clinical, biological, and virological monitoring was weekly for the first 3 months post‐KT. The turnaround time for serum creatinine measures was compatible with adjusting the VGCV dosage at each follow‐up visit. A real‐time quantitative PCR method in plasma samples was used to detect weekly DNAemia on the DxN VERIS system (Beckman Coulter, Brea, CA, USA) in the Marseille University Hospital Virology Laboratory. The lower limit of quantification was 350 IU/mL; below this, replication was deemed unquantifiable, and CMV infection was absent. CMV infection was defined as the presence of quantifiable DNAemia without clinical or biological signs of CMV disease. A CMV disease was defined as the presence of a quantifiable DNAemia associated with viral syndrome (i.e., fever > 38°C for more than 48 h, deterioration of general condition, leukopenia < 3.5 G/L, and/or thrombocytopenia < 100 G/L) or as tissue invasive disease (pulmonary, gastroenteritis, hepatic, neurological, retinal or any other organ dysfunction without any other identifiable cause) [10]. Preemptive treatment of CMV infection involved doubling the dosage of VGCV received in preventive doses. In case of CMV disease, curative treatment with ganciclovir (GCV) was administered for at least 21 days and stopped when two consecutive unquantifiable DNAemias or a negative CMV PCR were obtained. CMV PCR monitoring was performed weekly during curative treatment. At the end of the curative treatment, VGCV was resumed at a preventive dosage until the end of the prophylaxis period. The initial dosage for curative VGCV was also supposed to be adjusted according to the CG formula. In case of clinical suspicion, resistance to anti‐CMV drugs was researched in the National Reference Center in Limoges, France.

### 2.2 Study Design

All adult patients who received a KT between June 1, 2017 and June 1, 2020 in the Centre de Néphrologie et Transplantation Rénale in Marseille University Hospital, and who received anti‐CMV prophylaxis, were included in this retrospective single‐center case‐control study.

Patients presenting with CMV DNAemia during the first 3 months post‐KT despite VGCV prophylaxis were defined as cases. Each case was compared with four controls matched on sex, age, donor and recipient CMV IgG serostatus, and immunosuppressive induction treatment.

The data included in this study were anonymized, approved, and registered at the Health Data Portal of Assistance Publique—Hôpitaux de Marseille (PADS 21–62). The following were collected from medical records: donor‐related data (age, sex, CMV‐IgG serostatus, type of donor), and baseline patient‐related data (age, sex, nephropathy, diabetes, dialysis, previous KT, CMV‐IgG serostatus, anti‐HLA sensitization status, and calculated Panel Reactive Antibody [cPRA]). The immunosuppressive and anti‐infective treatments, cold ischemia time, delayed graft function (defined as the need for at least one dialysis session in the first week post‐KT), and number of HLA mismatches were recorded at the time of KT. After KT and at each visit, the following were noted: post‐transplantation diabetes mellitus (PTDM), daily dosages of prednisone and antimetabolites, CNI trough concentration, daily dosage of VGCV, and results of CMV PCRs. In the case of CMV DNAemia, CMV loads, presence of CMV disease, and elapsed times between the first DNAemia and preemptive or curative treatment onset were also recorded. If investigated, GCV resistance diagnosis was documented. Allograft loss and death were collected during the first year post‐KT.

**Figure 1:**
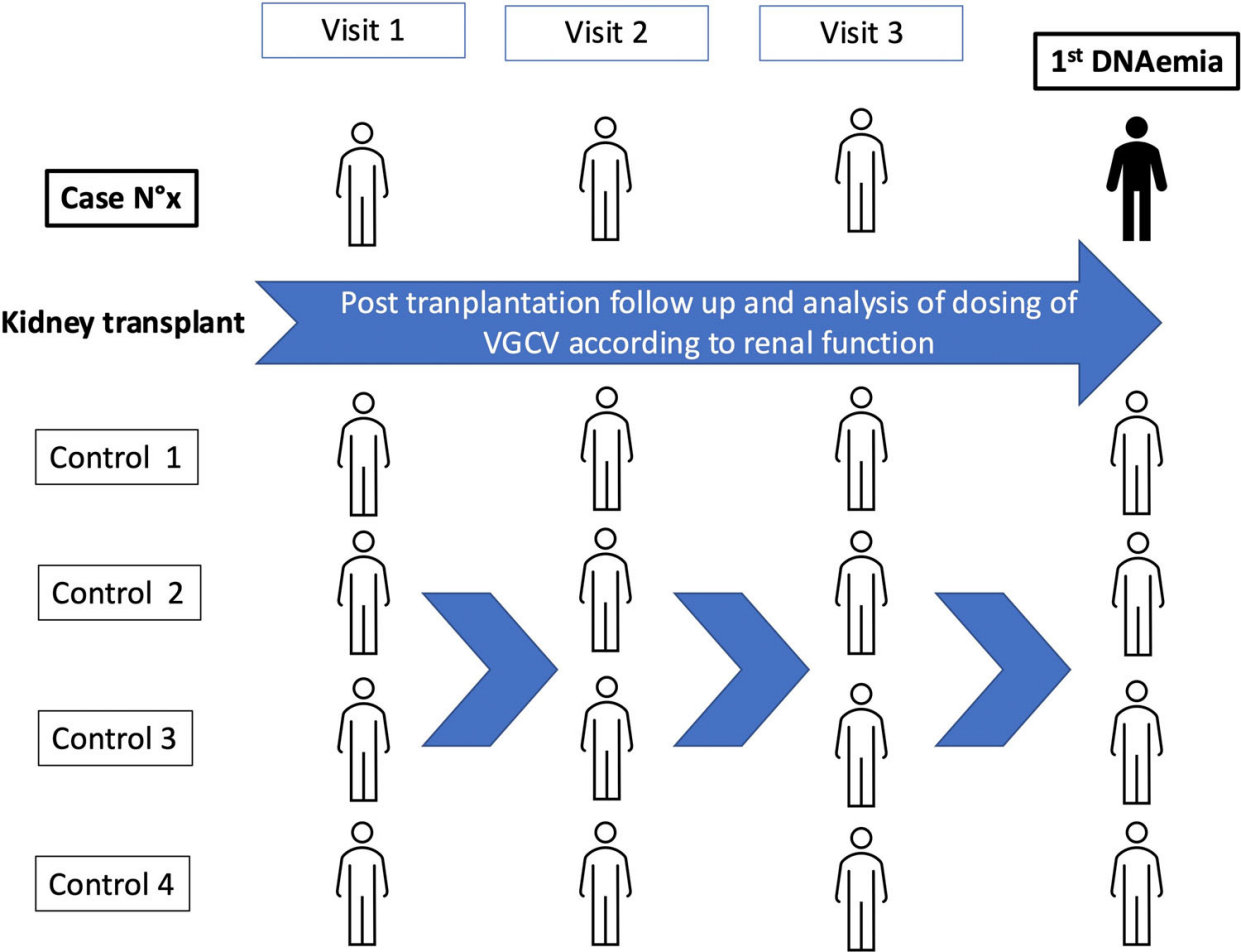
Period of analysis for valganciclovir dosing. The figure presents a case that had presented three negative CMV PCRs before detection of DNAemia after kidney transplant: We compared the received and the theoretical daily valganciclovir (VGCV) dosage in each case visit preceding the DNAemia and during an identical follow‐up period in the controls. We retrospectively calculated the estimated creatinine clearance by the Cockcroft–Gault formula, and the estimated glomerular filtration rate, indexed to body surface area or not, using the Chronic Kidney Disease Epidemiology equation, and then established the theoretical daily dosages of VGCV for each formula. At each visit, the effective dose received by the patient was categorized as “adjusted,” “underdosed,” or “overdosed” in comparison with the theoretical doses.

In the objective to analyze factors associated with DNAemia, we compared the received and the theoretical daily VGCV dosage in each case visit preceding the DNAemia and during an identical follow‐up period in the controls. We retrospectively calculated the estimated creatinine clearance by the CG formula, and the eGFR, indexed to body surface area (BSA) or not, using the 2009 CKD EPI equation, and then established the theoretical daily dosages of VGCV for each formula. At each visit, the effective dose received by the patient was categorized as “adjusted,” “underdosed,” or “overdosed” in comparison with the theoretical doses (Figure 1).

### 2.3 Statistical Analysis

Qualitative variables were described as counts and percentages, whereas quantitative variables were presented as means and standard deviations, or as medians and interquartile ranges (IQR). For the comparison of qualitative variables, the chi‐square test was used when appropriate, the Fisher test otherwise. For the comparison of the quantitative variables, the Student *t*‐test was used when appropriate, the Mann–Whitney test otherwise. Crude odds ratios (and their 95% confidence interval [CI]) were estimated using univariate conditional logistic regression models. The variables evaluated in the model included classic clinical and immunological patients’ and KT's characteristics, amongst them several already reported as associated with CMV infections: recipient nephropathy, diabetes and dialysis before KT, KT rank, HLA sensitization (anti‐HLA antibodies and cPRA), HLA mismatches [11], donor's type, sex and age, cold ischemia time [12], delayed graft function, and immunosuppressive regimen. The variables at DNAemia evaluated in the model were: estimated creatinine clearance, dosages of prednisone and antimetabolite, trough concentration of CNI, PTDM [13], and the rates of visits with VGCV underdosing since the KT calculated using the CG formula and the eGFR indexed to BSA or not.

The variables included in the multivariate model were the variables for which the *p* value is < 0.1 in the univariate model. Adjusted odds ratios (and their 95% CI) were estimated using a multivariate conditional logistic regression model. All tests were two‐sided, and for all analyses, a *p* value < 0.05 was considered statistically significant. All analyses were performed using IBM SPSS Statistics version 20.0 (SPSS Inc., Chicago, IL, USA) and R.

## 3 Results

### 3.1 Incidence of DNAemia Despite Prophylaxis and Main Characteristics of the Cases

**Figure 2:**
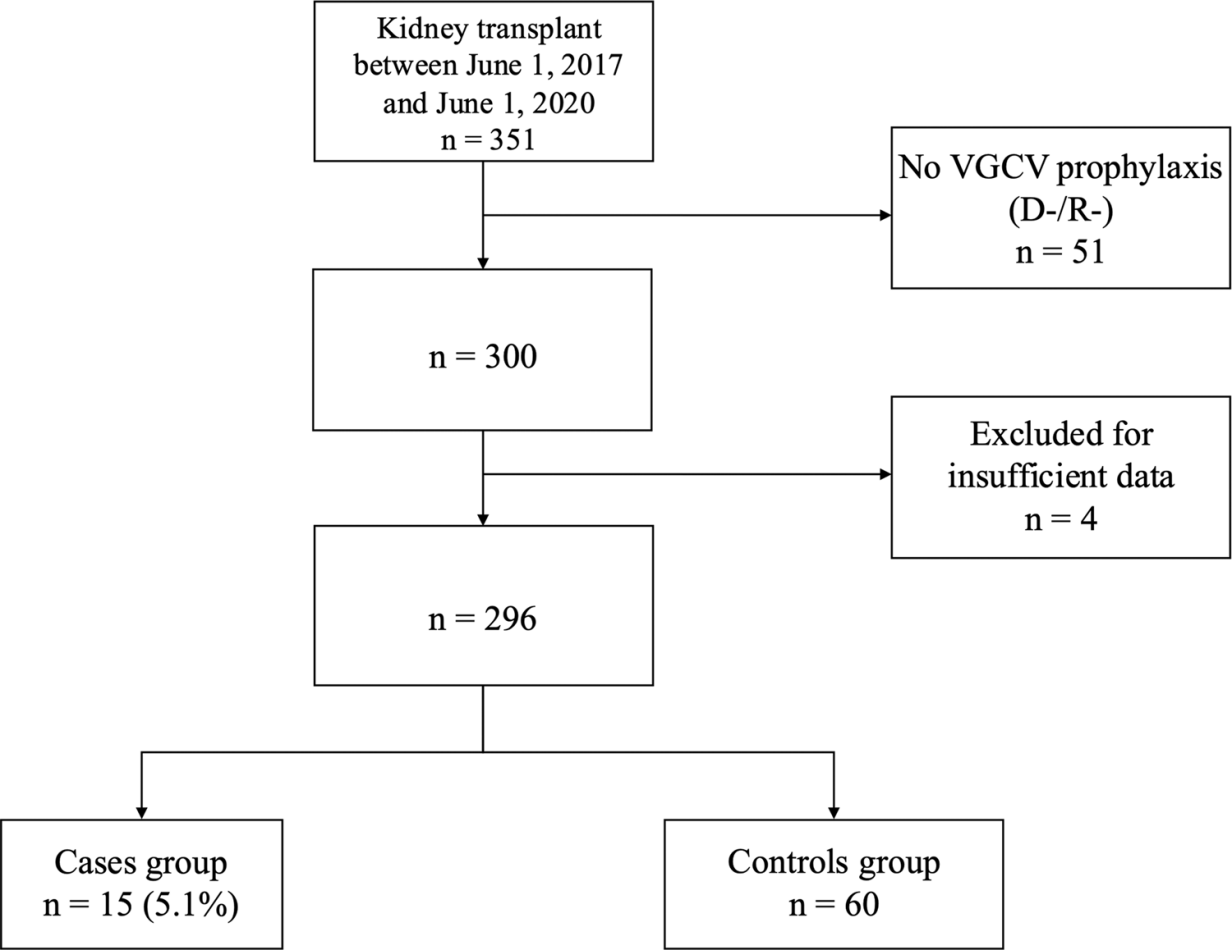
Flowchart. D−, donor with negative CMV‐IgG serology; R−, recipient with negative CMV‐IgG serology; VGCV, valganciclovir.

A total of 351 KT were performed during the study period in Marseille. As shown in Figure 2, 296 KTR under VGCV were included in the study. Despite VGCV prophylaxis, 15/296 (5.1%) KTR developed CMV DNAemia during the first 3 months following KT. Among the 15 cases, 12 (80%) were men, 2 (13%) presented a high risk of CMV infection, and 100% received antithymocyte globulin (Table 1). They were matched with 60 controls that received uninterrupted prophylactic VGCV treatment during the first 3 months after KT.

**Table 1:** Initial characteristics.

Characteristics at the time of KT	Cases (*n* = 15)	Controls (*n* = 60)	*p*
Recipients			
Male (%)	12 (80)	48 (80)	—
Mean age ± SD	61.40 ± 11.12	61.43 ± 10.67	—
Nephropathy			0.1
Diabetes (%)	0 (0)	9 (15)	
Nephroangiosclerosis (%)	3 (20)	9 (15)	
Genetic (%)	1 (6.7)	14 (23.3)	
Chronic glomerulonephritis (%)	3 (20)	8 (13.3)	
Chronic interstitial nephritis (%)	4 (26.7)	6 (10)	
Other (%)	0 (0)	3 (5)	
Unknown (%)	4 (26.7)	11 (18.3)	
Dialysis before KT (%)	14 (93.3)	55 (91.7)	0.84
CMV‐ IgG serology			
R+ (%)	13 (86.7)	52 (86.7)	—
R− (%)	2 (13.3)	8 (13.3)	—
KT rank			0.80
1 (%)	14 (93.3)	57 (95)	
2 (%)	1 (6.7)	3 (5)	
Preexisting diabetes (%)	3 (20)	18 (30)	0.41
Anti‐HLA antibodies > 5% (%)	2 (13.3)	9 (15)	0.85
cPRA, *n* (%)			0.97
< 20% (%)	14 (93.3)	39 (65)	
20%–85% (%)	0 (0)	17 (28.3)	
> 85% (%)	(6.7)	4 (6.7)	
Donors			
Male (%)	4 (26.7)	29 (48.3)	0.12
Mean age ± SD	61.67 ± 12.18	59.98 ± 13.17	0.54
Type of donor			0.91
Living (%)	1 (6.7)	4 (6.7)	
Brain‐dead (%)	11 (73.3)	48 (80)	
Uncontrolled cardiac death (%)	1 (6.7)	2 (3.3)	
Controlled cardiac death (%)	2 (13.3)	6 (10)	
Expanded criteria donor (%)	9 (60)	31 (51.7)	0.62
CMV‐ IgG serology			0.25
D+ (%)	12 (80)	39 (65)	
D− (%)	3 (20)	21 (35)	
Transplantations			
Mean cold ischemia time^a^ ± SD (h)	12.21 ± 4.35	13.02 ± 4.61	0.55
Mean HLA mismatches ± SD			
A	1.13 ± 0.64	1.25 ± 0.63	0.53
B	1.47 ± 0.64	1.42 ± 0.7	0.80
DR	0.73 ± 0.8	0.73 ± 0.63	1
DQ	0.53 ± 0.52	0.43 ± 0.53	0.48
Anti‐thymocyte globulin (%)	15 (100)	60 (100)	
Basiliximab (%)	0 (0)	0 (0)	
Tacrolimus (%)	8 (53)	31 (52)	0.88
Ciclosporine (%)	7 (47)	29 (48)	0.88
Mycophenolate mofetil (%)	10 (67)	34 (57)	0.31
Azathioprine (%)	5 (33)	26 (43)	0.31
Delayed graft function (%)	3 (20)	7 (12)	0.12
Primary non‐function (%)	1 (7)	0 (0)	—

Abbreviations: CMV, cytomegalovirus; cPRA, calculated panel reactive antibody; D+, donor with positive IgG CMV serology; D−, donor with negative IgG CMV serology; h, hours; HLA, human leukocyte antigen; KT, kidney transplant; R+, recipient with positive IgG CM; R−, recipient with negative IgG CMV; SD, standard deviation.

^a^ KT from living donors were excluded from the calculation of cold ischemia time.

The mean time to onset of DNAemia since KT was 34.6 ± 17.2 days, with extremes between 6 and 65 days (Table S1). At CMV DNAemia, no case had previously presented acute rejection. At the time of the DNAemia, the mean daily dosage of prednisone was 34.5 ± 21.6 mg, MMF 2000 ± 0 mg (*n* = 9), and azathioprine 129 ± 18.8 mg (*n* = 6). The mean trough concentration of tacrolimus was 10 ± 3.7 ng/mL in eight patients, while the mean trough concentration of ciclosporin was 192 ± 85 ng/mL in seven cases. Ten (66%) cases were diabetic, of which eight had PTDM. Regarding CMV prophylaxis at the time of DNAemia, the dosage of VGCV was 450 mg twice a week in three (20%) cases, 450 mg every other day in six (40%), and 450 mg daily in the last six (40%) patients. No patient received a full dose of 900 mg/day at the time of the DNAemia, whereas 19/60 (31.6%) of their respective controls received 900 mg/day of VGCV. Based on estimated creatinine clearance, 5/15 (33.3%) cases received an insufficient dosage, 6/15 (40%) received an appropriate dosage, and 4/15 (26.7%) received an excessive dosage of VGCV (Table S1). No interruption or reduction in the initial VGCV dosage had been prescribed before the CMV DNAemia.

### 3.2 Factors Associated With CMV DNAemia Despite Prophylactic Treatment With VGCV

At the time of KT, the characteristics of patients, donors, and KT procedures were comparable between the two groups (Table 1). At the time of CMV DNAemia, the mean daily dosages of immunosuppressive drugs and trough concentrations of CNIs were not different between the two groups (Table 2). In the cases group, there were a total of 50 visits preceding the 15 DNAemias (3.3 ± 1.9 visits per case). The rate of visits with VGCV underdosing was significantly higher in the cases group than in the controls group, whatever the formula used to estimate kidney function (Table 2). Overdosing based on estimated creatinine clearance was also observed during 6% and 21% of visits preceding DNAemia, in cases and controls, respectively.

**Table 2:** Characteristics after kidney transplantation.

	Cases (*n* = 15)	Controls (*n* = 60)	OR (95% CI)	*p*
PTDM at DNAemia, *n* (%)				0.15
No	6 (40)	36 (60)	1	
Yes	9 (60)	24 (40)	2.59 (0.71–9.4)	
Prednisone dosage at DNAemia(mg/day), mean ± SD	34.5 ± 21.6	34 ± 22.7	1 (0.94–1.07)	0.85
C0 Tacrolimus at DNAemia (ng/mL), mean ± SD	10 ± 3.7	8,8 ± 3.4	1.28 (0.92–1.76)	0.13
C0 Ciclosporine at DNAemia (ng/mL), mean ± SD	192.4 ± 85.4	200.6 ± 55.9	1 (0.98–1.02)	0.99
MMF at DNAemia dosage (mg/day), mean ± SD	2000 ± 0	1944 ± 199	0.97 (0.94–1.4)	0.41
Azathioprine dosage at DNAemia (mg/day), mean ± SD	129.1 ± 18.8	128 ± 19.9	1 (0.95–1.04)	0.9
Estimated creatinine clearance (mL/min)				
At D14^a^, mean ± SD	37.9 ± 19.5	51.8 ± 23.1	0.97 (0.94–1.001)	**0.05**
At DNAemia, mean ± SD	41.2 ± 18.8	50.6 ± 21.1	0.97 (0.93–1)	0.08
Rate of visits with underdosing VGCV before DNAemia				
Using CG	48%	14.5%	1.04 (1.01–1.06)	**0.003**
Using CKD EPI indexed on BSA	30%	5%	1.03 (1.008–1.057)	**0.01**
Using CKD EPI not indexed on BSA	35%	8%	1.03 (1.008–1.054)	**0.007**

*Note:* Bold values indicate statistically significant at *p* < 0.05.

Abbreviations: BSA, body surface area; C0, trough concentration; CG, Cockcroft–Gault; CI, confidence interval; CKD EPI, Chronic Kidney Disease Epidemiology; d, day; D14, Day 14; MMF, mycophenolate mofetil; OR, odds ratio; PTDM, post‐transplantation diabetes mellitus; SD, standard deviation; VGCV, valganciclovir.

^a^ Cases presenting DNAemia before day 14 were excluded from the analysis.

The creatinine clearance estimated by the CG formula at the time of DNAemia was higher, although not statistically, in the control group (50.6 mL/min) than in the cases group (41.2 mL/min) (OR: 0.97 [95% CI: 0.93–1; *p* = 0.08]). We retrospectively analyzed the evolution of the estimated creatinine clearance between day 14 post‐KT in 13 cases and their controls that had not at that time presented DNAemia, and the day of CMV DNAemia (Table 2). We observed a worse renal function in the cases group compared to the controls group at day 14 (37.9 mL/min vs. 51.8 mL/min, *p* = 0.05) and an improvement of renal function in cases, on DNAemia day (41.3 vs. 50.6 mL/min, *p* = 0.08).

The OR for developing CMV DNAemia under prophylaxis with VGCV underdosing was 1.04 (95% CI: 1.012–1.06; *p* = 0.003), 1.03 (95% CI: 1.008–1.057; *p* = 0.01), and 1.03 (95% CI: 1.008–1.054; *p* = 0.007), per percent of visits when using the CG, CKD EPI indexed and not indexed on BSA formulas, respectively.

The multivariate analysis showed that the rate of visits with an underdosing of VGCV, whatever the equation used to estimate the kidney function, was an independent risk factor of DNAemia occurrence with an OR of 1.03–1.04 (Table 3).

**Table 3:** Multivariate analysis of factors associated with CMV DNAemia.

	Cases	Controls	Adjusted OR (CI 95%)	*p*
Estimated creatinine clearance				
At D14^a^ (mL/min) mean	37.9	51.8	0.97 (0.91–1.032)	0.36
At DNAemia (mL/min) mean	41.2	50.6	0.97 (0.93–1.014)	0.19
Rate of visits with underdosing VGCV before DNAemia				
Using CG	48%	14.5%	1.04 (1.01–1.06)	**0.004**
Using CKD EPI indexed on BSA	30%	5%	1.03 (1.01–1.05)	**0.008**
Using CKD EPI not indexed on BSA	35%	8%	1.03 (1.01–1.06)	**0.004**

*Note:* Bold values indicate statistically significant at *p* < 0.05.

Abbreviations: BSA, body surface area; CG, Cockcroft–Gault; CI, confidence interval; CKD EPI, Chronic Kidney Disease Epidemiology; D14, Day 14; OR, odds ratio; VGCV, valganciclovir.

^a^ Cases presenting DNAemia before day 14 were excluded from the analysis.

### 3.3 Clinical Evolution of Patients With DNAemia Despite Prophylaxis

Among the 15 cases, 5 patients (33%) received curative treatment with IV GCV for CMV disease, 8 (53%) received preemptive treatment for CMV infection, and 2 (13%) were not treated (Table S2). The retrospective analysis of these two individuals showed that patient no. 5 presented an initial low viral load of 690 IU/mL, then negative PCR 1 day later, and that patient no. 8 presented a maximal viral load of 464 IU/mL, then negative PCR 9 days later. The median initial viral load for cases was 972 IU/mL (IQR, 600–1632). Among the 13 treated cases, 3 patients were treated from the first DNAemia, and 10 patients after controlling positive PCR that showed an approximately 10‐fold increase in viral load. The mean time between the first DNAemia and the introduction of a preemptive treatment was 8.9 ± 7.7 days. At this time, the median viral load was 3439 IU/mL (IQR, 2434–6213). Five patients developed CMV disease despite preemptive treatment and received curative treatment with IV GCV. The median viral load at the time of IV GCV introduction was 32 127 IU/mL (IQR, 29 646–34 255). The mean elapsed time between the IV GCV curative treatment onset and the first DNAemia was 53.8 ± 32.1 days, with extremes between 15 and 90 days. Manifestations of CMV disease were: thrombocytopenia (patient no. 1, 33 days after the first DNAemia), altered general condition (patient no. 4, 15 days after the first DNAemia), leukopenia (patients no. 6 and 10, 90 and 40 days, respectively, after the first DNAemia), and pneumonia (patient no. 12, 83 days after the first DNAemia).

The mean duration of DNAemia was 26.7 ± 16.1 days in 11/15 cases, with extremes between 1 and 54 days. We could not calculate it *stricto sensu* in the case of patients no. 6, 10, 12, and 4. In the first three cases, DNAemia never cleared or never became unquantifiable on two consecutive samples, and patient no. 4 died during CMV disease. For these four cases, we diagnosed CMV resistance to GCV at 82 ± 14.8 days after the first DNAemia. Mutations were found in UL97 in patients no. 4, 6, and 10, and in UL54 in patient no. 12. Two of four cases were at high risk of CMV infection (D+/R−). Infections resolved in patients no. 6, 10, and 12 after IV Foscavir treatment.

Two patients (nos. 1 and 14) presented a DNAemia recurrence during the follow‐up, respectively 70 and 136 days after transplantation, but with a favorable evolution.

All the patients except one were alive at the end of the 1‐year follow‐up (Table S2). Patient no. 4 received a VGCV preemptive treatment 8 days after the first DNAemia, then a curative treatment, 6 days later. He died from unexpected cardiac arrest after urological surgery, 78 days after the first DNAemia. His CMV IgG serostatus was negative, and 72 days after the first DNAemia, a mutation conferring resistance to GCV was found after the patient's death. The last measured viral load was 46 058 IU/mL, 2 days before death and after 61 days of GCV treatment. Two patients lost their grafts, patient no. 7 due to transplant venous thrombosis 1 day after KT and 5 days before the diagnosis of CMV infection, and patient no. 9, due to multifactorial dysfunction 65 days after KT, following an initial graft infarction.

## 4 Discussion

In this study, we aimed to determine the incidence of CMV DNAemia in the first 3 months following KT in the context of universal VGCV prophylaxis, and to identify the associated factors. In a cohort of 296 KTR over a 3‐year period and using routine weekly CMV PCR follow‐up, the incidence of CMV DNAemia was 5.1%. This is quite close to that reported in previous studies under VGCV prophylaxis, where it ranged from 1.9% to 6% [6, 14, 15, 16]. Differences in inclusion criteria and frequency of CMV PCR monitoring could explain some of the disparities between studies. CMV PCR was monitored every 2 weeks during the first 3 months following KT in the studies by Wéclawiak et al. and Schaenman et al. [6, 14]. The CMV DNAemia incidence was 1/51 (1.9%) and 16/715 (2.2%), respectively. Schaenman et al. focused solely on KTR receiving a transplant from a CMV seropositive donor [6]. An Australian prospective trial including 50 KTR reported a 6% incidence in the first 3 months after KT, when CMV PCRs were performed at the sole discretion of the nephrologists [16]. Surprisingly, Trevillyan et al. reported a 50% incidence over the first 6 months post‐transplantation in a small population of 22 liver and KT recipients receiving VGCV prophylaxis. The duration of CMV prophylaxis and the CMV PCR monitoring modalities were not specified [7].

Under well‐conducted VGCV prophylaxis, we could expect a zero incidence of CMV DNAemia. In the present study, where the 15 cases were matched to 60 controls for sex, age, CMV IgG serostatus of both donors and recipients, and immunosuppressive induction treatment, univariate and multivariate analyses showed that the rate of visits (in other words, the time spent) with VGCV underdosing was a risk factor for the occurrence of CMV DNAemia. Retrospective assessment of kidney function according to different formulas showed that the rate of visits with VGCV underdosing in cases was always significantly higher than in controls, whatever the formula used. Furthermore, we showed an improvement of renal function in cases between day 14 post‐KT and on DNAemia day. Taken together, these data suggest that renal function was not assessed at each post‐transplant visit and that the VGCV dosage was not adjusted, leading to underdosing. The rate of visits with VGCV underdosing in cases was 48%, 35%, and 30%, using the formulas for CG, not BSA‐indexed CKD EPI, and BSA‐indexed CKD EPI, respectively, meaning that underdosing is missed in 13%–18% of visits when estimated GFR is used rather than the CG formula. Another explanation for the underdosing could be concern about the toxicity of VGCV, particularly when estimated creatinine clearance approaches a threshold value that theoretically indicates the need for a dose increase.

Our study raises, as most studies do, the issue of VGCV dosage as key to inefficient prevention of CMV infection/diseases as a result of underdosing related to the use of kidney function assessment formulas other than CG [7, 14, 17], or as a result of not giving the maximal dose of VGCV (900 mg/day) for whatever reason [6, 18]. On the contrary, few studies have reported that VGCV underdosing has no impact on infections, but the conditions for monitoring PCR and renal function were less stringent than in our study [9, 19]. Some centers using VGCV low‐dose prophylaxis in the hope of improving tolerability showed similar CMV outcomes. However, the most recent international guidelines advise against “mini‐dosing” because these studies were not powered to show different outcomes [4].

The constraint of adjusting the VGCV dosage to renal function, compared with the easiest use of letermovir that does not need it, may have contributed to the results of a randomized noninferiority, Phase 3 trial comparing letermovir and VGCV among adult CMV‐seronegative KTR who received an organ from a CMV‐seropositive donor. No participants who received letermovir versus five participants (1.7%) who received VGCV developed CMV disease through week 28 [20]. The latest consensus conferences for the management of CMV infections in solid organ transplantation recommend the use of creatinine clearance estimated by the CG equation for dose adjustment of VGCV to kidney function [4]. To ensure that the VGCV dosage is systematically adjusted at each visit, an automatic calculation program for estimated creatinine clearance could be easily achieved in the post‐transplant follow‐up form.

To date, measuring blood GCV concentration is not recommended to monitor CMV infection treatment until robust data are available to define the validity of therapeutic drug monitoring [4]. No association was found between trough blood concentration of GCV and CMV DNAemia [7], and in a recent systematic review, data were inadequate to establish a target GCV trough blood concentration in CMV infection prophylaxis in solid organ transplantation [21].

In another study, authors suggested that the immune dysfunction associated with impaired kidney function contributed to the development of DNAemia [6], but we observed that kidney function was not an independent factor associated with CMV DNAemia based on multivariate analysis in our study.

Resistance of CMV to GCV could be a factor in the occurrence of DNAemia under VGCV. In our study, 4/15 (27%) of patients presented CMV disease with CMV resistant to GCV. We cannot determine if resistance was present from the onset or emerged later, because this was not systematically tested. Two of the four resistant cases had received underdosed VGCV for 75% of consultations preceding DNAemia. VGCV underdosing has already been described as a risk factor for the emergence of CMV resistant to GCV in recipients of kidney, hepatic, or pancreatic transplants [16, 22, 23].

Weekly monitoring of CMV PCR allows for preemptive treatment of CMV infection, before clinical and biological signs of CMV disease appear. In the context of VGCV prophylaxis, questions of diagnosis and treatment of excess CMV infection may arise. Positive predictive value of VERIS PCR to diagnose active CMV replication was 100% for a threshold of 17 000 IU/mL. In our study, only one case had > 17 000 IU/mL before preemptive treatment, and two cases were not treated because of the spontaneous disappearance of the DNAemia at the next consultation. Furthermore, spontaneous viral clearance has been described in the absence of CMV infection prophylaxis or treatment. It was reported that after a period of prophylaxis with VGCV for 3 or 6 months after KT, 36 of the 95 (37.8%) CMV DNAemia patients showed spontaneous viral clearance with an average duration of 78 ± 137 days. Associated factors were initial viral load < 2.75 log10 IU/mL and no increase by a factor of 10 in the following 4 investigations [24]. In the latest consensus conferences for the management of CMV infections in solid organ transplantation, no threshold has been set for starting treatment during a preemptive regimen [4]. However, it is specified that an increase in viral load greater than 0.5 Log10 or 0.7 Log10 for initial values < 3 Log10 between two samples should be considered significant.

Our study has limitations as it is monocentric and retrospective. The rarity of CMV DNAemia under VGCV prophylaxis prevented the identification of other factors than underdosing of VGCV, and we did not evaluate the cost of our strategy.

In conclusion, in high or intermediate risk of CMV infection, KT under prophylaxis and weekly monitoring of CMV PCR, we found an incidence of DNAemia of 5.1% in the first 3 months post‐KT. We showed that the time during which VGCV was underdosed was a risk factor for CMV DNAemia. The absence of systematic adjustment of VGCV dosages or the use of different formulas other than CG to assess kidney function and adjust VGCV dosages during follow‐up visits might have explained underdosing and been complicated by CMV disease and resistance of CMV to GCV.

## Author Contributions

V.M. and P.‐A.R. designed the study and drafted the manuscript. P.‐A.R. collected the data. N.R. supervised the statistical analysis. J.S., C.Z., M.G., P.B., and T.L. critically reviewed the manuscript. All authors contributed to the article and approved the submitted version.

## Conflicts of Interest

The authors declare no conflicts of interest.

## Supporting information


**Supplementary Table 1:** Characteristics of cases during the first episode of DNAemia during valganciclovir prophylaxis.

**Supplementary Table 2:** Evolution of the cases.

## Data Availability

The data that support the findings of this study are available from the corresponding author upon reasonable request.
