# Supplementary material for: Valganciclovir Underdosing Is Associated With Cytomegalovirus DNAemia During Universal Prophylaxis: A Real‐Life Case Control Retrospective Study
Source: J Med Virol. 2025 Oct 24;97(11):e70666. doi: 10.1002/jmv.70666 (PMC12551442; doi:10.1002/jmv.70666)
Supplement: Supplementary file 1 — Supplementary Table 1: Characteristics of cases during the first episode of DNAemia during valganciclovir prophylaxis. [file JMV-97-e70666-s001.docx]

**Supplementary Table 1. Characteristics of cases during the first episode of DNAemia during valganciclovir prophylaxis**

| Case | Sex | Age (y) | D/R  serostatus | Duration between DNAemia and KT (d) | CG Estimated creatinine clearance  (mL/min) | Steroids (mg/d) | C0 CNI (ng/mL) | Antimetabolite (mg/d) | Dosing of VGCV | Manufacturer- recommended dose |
| --- | --- | --- | --- | --- | --- | --- | --- | --- | --- | --- |
| 1 | M | 53 | D+/R+ | 6 | 24 | 90 | T: 9.7 | MMF: 2000 | 450mg/48h | 450mg twice a week |
| 2 | M | 56 | D+/R+ | 15 | 73 | 60 | T: 17.3 | MMF: 2000 | 450mg/24h | 900mg/24h |
| 3 | M | 53 | D+/R+ | 48 | 69 | 20 | C: 176 | MMF: 2000 | 450mg twice a week | 900mg/24h |
| 4 | M | 66 | D+/R- | 53 | 46 | 17,5 | C: 217 | A: 100 | 450mg/24h | 450mg/24h |
| 5 | M | 77 | D+/R+ | 39 | 33 | 25 | C: 123 | A: 125 | 450mg/48h | 450mg/48h |
| 6 | W | 63 | D+/R- | 43 | 39 | 30 | T: 5.6 | MMF: 2000 | 450mg/48h | 450mg/48h |
| 7 | M | 75 | D+/R+ | 6 | 0 | 70 | C: 370 | A: 125 | 450mg twice a week | 100 mg after HD |
| 8 | W | 46 | D-/R+ | 21 | 37 | 30 | T: 11.9 | MMF: 2000 | 450mg/24h | 450mg/48h |
| 9 | M | 70 | D+/R+ | 34 | 26 | 25 | C: 134 | A: 150 | 450mg/48h | 450mg/48h |
| 10 | W | 68 | D+/R+ | 38 | 52 | 20 | C: 190 | A: 150 | 450mg/24h | 450mg/24h |
| 11 | M | 54 | D-/R+ | 34 | 39 | 30 | T: 9.7 | MMF: 2000 | 450mg/24h | 450mg/48h |
| 12 | M | 79 | D+/R+ | 65 | 40 | 20 | C: 137 | A: 125 | 450mg/48h | 450mg/24h |
| 13 | M | 65 | D+/R+ | 54 | 67 | 20 | T: 7 | MMF: 2000 | 450mg/24h | 900mg/24h |
| 14 | M | 45 | D-/R+ | 33 | 40 | 40 | T: 6.2 | MMF: 2000 | 450mg twice a week | 450mg/24h |
| 15 | M | 51 | D+/R+ | 30 | 34 | 20 | T: 12.2 | MMF: 2000 | 450mg/48h | 450mg/48h |

y: year, D: Donor, R: Recipient, KT: Kidney transplant, d: day, CG: Cockcroft-Gault, C0 CNI: Trough level of calcineurin inhibitor, VGCV: Valganciclovir, M: Man, W : Woman, D+: Donor with positive IgG CMV serology, D-: Donor with negative IgG CMV serology, R+: Recipient with positive IgG CMV, R-: Recipient with negative IgG CMV serology, T: Tacrolimus, C: Ciclosporine, MMF: Mycophenolate mofetil, A: Azathioprine, HD: Hemodialysis
