# Supplementary material for: Valganciclovir Underdosing Is Associated With Cytomegalovirus DNAemia During Universal Prophylaxis: A Real‐Life Case Control Retrospective Study
Source: J Med Virol. 2025 Oct 24;97(11):e70666. doi: 10.1002/jmv.70666 (PMC12551442; doi:10.1002/jmv.70666)
Supplement: Supplementary file 2 — Supplementary Table 2: Evolution of the cases. [file JMV-97-e70666-s002.docx]

**Supplementary Table 2. Evolution of the cases**

| Case | Initial DNAemia (IU/mL) | DNAemia at treatment initiation (IU/mL) | Duration before treatment (d) | CMV infection or disease | Duration of DNAemia* (d) | Test for resistance | Duration before resistance diagnosis (d) | Mutation conferring resistance | Evolution |
| --- | --- | --- | --- | --- | --- | --- | --- | --- | --- |
| 1 | 979 | 4717 | 7 | Disease | 54 | No | - | - | Resolved |
| 2 | 1504 | 24066 | 14 | Infection | 36 | No | - | - | Resolved |
| 3 | 1632 | 4987 | 7 | Infection | 21 | No | - | - | Resolved |
| 4 | 600 | 6213 | 8 | Disease | - | Yes | 72 | UL97 Ala594Val | Died at D131 |
| 5 | 690 | - | - | - | 1 | No | - | - | Resolved |
| 6 | 2403 | 12047 | 7 | Disease | - | Yes | 94 | UL97 A594P | Resolved |
| 7 | 863 | 3439 | 7 | Infection | 26 | No | - | - | Graft loss at D1 |
| 8 | 464 | - | - | - | 9 | No | - | - | Resolved |
| 9 | 1953 | 1953 | 0 | Infection | 35 | No | - | - | Graft loss at D65 |
| 10 | 465 | 2478 | 27 | Disease | - | Yes | 67 | UL97 L595S | Resolved |
| 11 | 2267 | 2267 | 0 | Infection | 14 | No | - | - | Resolved |
| 12 | 433 | 3053 | 14 | Disease | - | Yes | 96 | UL54 T503I/T | Resolved |
| 13 | 972 | 972 | 0 | Infection | 28 | No | - | - | Resolved |
| 14 | 669 | 2434 | 7 | Infection | 35 | No | - | - | Resolved |
| 15 | 391 | 13814 | 18 | Infection | 35 | No | - | - | Resolved |

* In patient n°6, monitoring was stopped 153 days after the first DNAemia due to stagnation of DNAemia closed to the limit of quantification. Patient n°10 presented an unquantifiable DNAemia 98 days after the first one; 7 days later, DNAemia was 379 IU/mL, then monitoring was interrupted. Patient n°12 presented an unquantifiable DNAemia 148 days after the first one; 7 days later, DNAemia was 562 IU/mL, then monitoring was interrupted.

d: Day, CMV: Cytomegalovirus, D131: Day131
